# Detection of prediabetes and undiagnosed type 2 diabetes in underserved communities using community pharmacy point‐of‐care testing: A prospective feasibility study

**DOI:** 10.1111/dme.70368

**Published:** 2026-05-15

**Authors:** Andrew Radley, Lewis Beer, Albert Farre, Petra Rauchhaus, George Thom, Calum Sutherland

**Affiliations:** ^1^ Faculty of Health University of Dundee, Airlie Place Dundee UK; ^2^ Tayside Clinical Trials Unit, Ninewells Hospital and Medical School University of Dundee Dundee UK; ^3^ Public Health Directorate NHS Tayside, Ninewells Hospital and Medical School Dundee UK

**Keywords:** community pharmacy, diabetes, missingness, point‐of‐care, prevention, primary care

## Abstract

**Aims:**

Early detection of type 2 diabetes (T2D) and prediabetes is critical to reduce disease burden, yet disadvantaged populations often remain undiagnosed. We assessed a pharmacy‐based pathway using verified point‐of‐care HbA1c testing to identify prediabetes and enable engagement with a digital prevention programme and established the detection rates for undiagnosed T2D.

**Methods:**

We conducted a prospective feasibility study across 16 community pharmacies in NHS Tayside, Scotland (Feb 2024–Jan 2025). Adults at high risk of T2D, identified using the Diabetes UK ‘*Know Your Risk*’ tool, were offered HbA1c testing. Participants with HbA1c 42–47 mmol/mol (6.0–6.4%) were referred directly to a digital diabetes prevention programme (DDP); those with HbA1c ≥48 mmol/mol (6.5%) were advised to attend their GP. Comparator data were drawn from GP patients identified for engagement with the same DPP.

**Results:**

Of 499 pharmacy participants, 75 (15%) had prediabetes; 38 (51%) enrolled in DPP. Nineteen (4%) had HbA1c ≥48 mmol/mol (6.5%); 17 attended GP. Compared with GP‐identified prediabetes (951 patients; 212 enrolled, 32%), pharmacy‐based recruitment doubled DDP engagement (RR 2.08, 95% CI 1.61–2.67). Pharmacy recruited relatively younger participants from more deprived backgrounds (63% SIMD 1–2 vs. 33% in GP cohort).

**Conclusion:**

Community pharmacies provided effective, equitable access to HbA1c testing and diabetes prevention, identifying individuals with prediabetes and undiagnosed T2D being missed by current pathways and doubling engagement of this underserved population with DPP. Pharmacy‐based screening offers a scalable opportunity to reduce health inequalities and expand primary care capacity.

**What's new?**

**What is already known on this topic?**

- Many people must live with type 2 diabetes or prediabetes for many years prior to diagnosis and access to care
- Health outcomes improve significantly with early detection and intervention
- Poor outcomes are particularly prevalent in those living in socioeconomically disadvantaged communities who suffer higher rates of undiagnosed type 2 diabetes
- Community pharmacies offer accessible locations for opportunistic diabetes risk and glycaemia assessment.
- Evidence on real‐world performance of clinically relevant point‐of‐care HbA1c testing in pharmacies remains limited.

**What this study adds?**

- Community pharmacies can accurately identify prediabetes and previously undiagnosed diabetes using verified HbA1c point‐of‐care testing.
- The pharmacy pathway identified proportionally more individuals in need of available care packages from socio‐economically deprived areas compared with GP‐based identification.
- Direct pharmacy referral more than doubled engagement with a digital diabetes prevention programme compared with the GP‐identified cohort.

**What are the implications of our findings?**

- Pharmacy‐based HbA1c testing discovers relatively high levels of asymptomatic type 2 diabetes in underserved communities and increases the reach of type 2 diabetes prevention services, particularly for those living with high deprivation. In both cases, this provides earlier detection and referral to care for the hardest to reach but those most at risk of long‐term health problems.
- Embedding verified point‐of‐care testing with direct referral pathways will improve early detection of type 2 diabetes and prediabetes, whilst increasing uptake of preventive interventions, especially amongst people not currently engaging with general practice services.
- This model is feasible, scalable and complementary to primary care capacity. Integrating community pharmacies more closely with the general practice network may improve engagement of those currently missing from care.

## 1 INTRODUCTION

The number of adults with Type 2 Diabetes (T2D) is expected to increase from 415 million to 642 million between 2015 and 2040.^1^ Many cases (~175 million) are currently undiagnosed, with 230 million people exhibiting non‐diabetic hyperglycaemia.^2^ In Scotland, T2D prevalence increased from 190,772 in 2008 to 267,615 in 2018^3^ with a greater burden falling on disadvantaged communities.

The number of deaths where T2D was recorded as a contributory factor also increased, rising from 58.3 per 100,000 in 2011/12 to 120.8 in 2023/24, with the rates for males (155.3 per 100,000) much higher than for females (93.9 per 100,000).^4^ This has led to population‐based screening programmes such as the diabetes screening component of the National Cardiovascular Risk Assessment Programme in England, to achieve earlier T2D diagnosis and implement care through general practices to prevent progression to micro‐ and macro‐vascular complications.^5^ Screening for high‐risk groups is currently not performed in Scotland.

T2D has a prodromal stage usually referred to as prediabetes, where individuals exhibit intermediate hyperglycaemia. Prediabetes represents an opportunity for early intervention to prevent progression to T2D and improve longer term outcomes. The challenge is to deliver early detection and intervention to these individuals at imminent risk of T2D, without overburdening primary care.^6^ T2D remission can be achieved with significant reduction in body adiposity leading to weight loss.^7^ Validated structured weight management programmes with significant success in delivering remission are now available in the NHS.^8^ Similarly, weight loss is proven to prevent, or delay progression to, T2D even in those at highest risk of developing the disease. Such interventions can be delivered in primary care and people can maintain weight reduction for significant periods,^9^ with weight loss in at‐risk individuals contributing to reduction in T2D incidence.

Many governments have policies to increase referrals of those at highest risk, to services that might trigger early adoption of weight loss strategies, (e.g. social prescribing, remote digital consultations and health coaching).^7^ This requires establishing routine and accurate early detection for large numbers of ‘at‐risk’ individuals.^10^ This could be done through risk assessment tools (now commonly available), to guide assessment by health professionals and for public education.^11^ Early detection and intervention of those who may benefit most (e.g. fast progressors) should ensure best use of limited resources.^5^ Community pharmacies are present in each town and village and have significant reach into disadvantaged populations, where there is a higher proportion of at‐risk individuals.^12^

The diagnosis of T2D, or prediabetes, historically relied upon measurement of plasma glucose levels.^13^ In community pharmacy settings, utilising this measure is complicated by the prandial status of the individual, as glucose homeostasis is affected by dietary intake.^14^ The alternative measure of HbA1c quantification is more suited to opportunistic detection, reflecting glycaemia status over the last 90 days.^15^ Typically measurement of HbA1c relies upon central laboratories, with the requirement for planned phlebotomy. However, measurement of HbA1c using validated point‐of‐care (POC) testing is now possible.^16^ We have established that the Abbott Afinion instrument provides results precise enough for clinical decision making in a community pharmacy environment.^17^

Community testing of HbA1c to accepted clinical standards facilitates access to individuals from populations historically poor at engaging with primary care: representing a strategy to combat health inequalities.^18^ Opportunistic testing in such communities should permit those living with undiagnosed diabetes, or prediabetes, to access care without screening by GP practices.

Our paper demonstrates the feasibility of implementing a novel community pharmacy pathway using verified HbA1c POC testing to enhance access to T2D prevention programmes for individuals with prediabetes situated within areas of the lowest socio‐economic deprivation index in Scotland. The engagement with these programmes is two‐fold higher for individuals recruited by community pharmacy compared to current recruitment approaches through GP practices. An added benefit of this pathway is the serendipitous detection of undiagnosed T2D in this population who normally engage relatively poorly with primary care.

## 2 MATERIALS AND METHODS

### 2.1 Study design

We conducted a prospective, non‐randomised feasibility study with an embedded process evaluation. The study was designed to assess the implementation of a community pharmacy pathway for detection of prediabetes and referral into a digital diabetes prevention programme (DDP).

The study was reviewed by the Tayside Medical Science Centre Governance service and assessed to be a service improvement project, not requiring ethical review. Caldicott Guardian approval and local service evaluation permissions were obtained from NHS Tayside. Ethics permission for the staff interviews was granted by the University of Dundee Research Ethics Committee (UoD‐SMED SLS‐Staff‐2024‐24‐43).

### 2.2 Setting

The study was undertaken in NHS Tayside, Scotland, between February 1, 2024, and January 31, 2025. Sixteen community pharmacies serving approximately 78,000 patients were recruited. Comparator data were obtained using prediabetes patients identified by 59 GP practices and offered a place in the same DDP within the same health board (caseload ~448,000).^19^ The communities served by pharmacies and general practices are classified as large urban, small urban and other urban areas and included communities experiencing a high degree of socio‐economic disadvantage, as classified by the Scottish Index of Multiple Deprivation (SIMD).

### 2.3 Participants

Eligible participants were adults (≥18 years) attending participating pharmacies who scored ≥16 on the Diabetes UK ‘*Know Your Risk*’ tool. All participants provided written informed consent. Exclusion criterion was inability to provide consent. Comparator participants were those identified to the DDP by general practitioners during the same period.

Individuals who were eligible but did not proceed to screening or HbA1c testing typically declined due to limited time, unwillingness to have a finger‐prick test, or no perceived need for further assessment. Reasons were not systematically captured, reflecting the pragmatic nature of the feasibility study.

### 2.4 Procedures

Each participating pharmacy was provided with resources to assess T2D risk status and test HbA1c on‐site. Pharmacy teams attended a training event and were provided with standard operating procedures and learning materials to support reliable operation of the instrument. On‐going support was available through the study team. Pharmacies were provided with posters and leaflets to publicise the service.

The point‐of‐care instrument used was the Abbott Afinion 2,^20^ which is a fully automated device that utilises Boronate Affinity Chromatography to measure glycated haemoglobin (HbA1c) in a 1.5 μL sample of venous or capillary whole blood. The verification of the instruments was performed in line with the NHS Tayside Blood Sciences Department Standard Operating Procedure, for the validation and verification of processes.^17^

**FIGURE 1:**
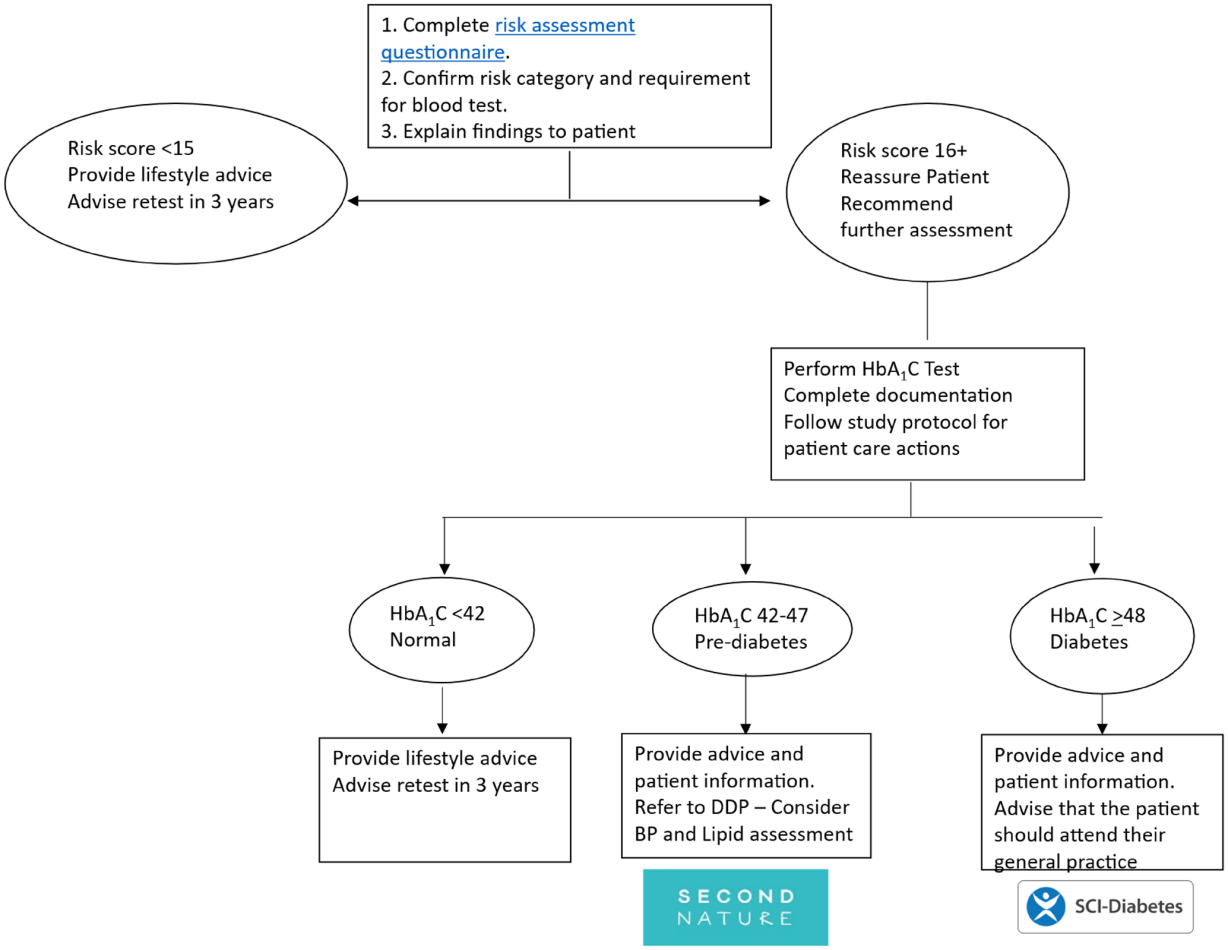
Pharmacy HbA1c pathway.

People using the pharmacy were invited to engage with the pathway (Figure 1: Pharmacy HbA1c Pathway) and informed consent obtained before initiating the pathway:

- Potential participants in pharmacies answered the questions contained in the Diabetes UK ‘*Know Your Risk*’ assessment (https://riskscore.diabetes.org.uk/start) to establish their risk of T2D
- All consenting patients who scored 16+ using the tool were offered a POC HbA1c test. A capillary blood sample was taken from the finger to test on the Afinion 2.
- Consenting patients who tested between 42 and 47 mmol/mol (6.0–6.4%) (prediabetes) were referred to a Digital Diabetes Prevention Programme (DDP) ‘*Second Nature’* (https://www.secondnature.io), provided through NHS Tayside.^21^ A copy of their assessment was sent to their GP.
- Consenting patients who tested 48 mmol/mol (6.5%) or more were advised to attend their GP for further assessment of possible T2D. A copy of their assessment was sent to their GP.

### 2.5 Variables

- Exposure: HbA1c tested at community pharmacy pathway vs. general practice identification of prediabetes
- Primary outcome: Detection of prediabetes (HbA1c 42–47 mmol/mol) (6.0–6.4%) and subsequent enrolment in the DDP.
- Secondary outcomes: Detection of previously undiagnosed T2D (HbA1c ≥48 mmol/mol) (6.5%), engagement with GP follow‐up and predictive performance of risk‐assessment tools (BMI, waist‐to‐height ratio, ‘*Know Your Risk*’ score).
- Covariates: Age, sex, smoking status, prescription of antihypertensives, Scottish Index of Multiple Deprivation (SIMD) quintile, ethnicity

### 2.6 Data sources and measurement

HbA1c was measured using the Abbott Afinion 2 point‐of‐care analyser, with verified performance using paired samples against NHS Tayside Blood Sciences protocols. Risk scores and anthropometric data were collected by pharmacy staff using standardised case report forms. Comparator data were extracted from NHS datasets and SCI‐Diabetes. Prediabetes diagnosis and DDP engagement were confirmed via CHI number linkage.^22^ Anthropometric data for the people engaged by their general practitioners was not available to the study.

### 2.7 Bias

Selection bias was minimised by offering the pathway to all eligible pharmacy attendees. Information bias was reduced through staff training and standard operating procedures. Comparator data were population‐based, reducing ascertainment bias.

### 2.8 Study size

We aimed to recruit 40–60 participants per pharmacy (3–5 tests per month), yielding ~500 participants overall. Comparator group size was determined by all individuals with prediabetes identified by NHS Tayside GP practices for engagement with the DDP during the study period (*n* = 951).

### 2.9 Quantitative variables

HbA1c was analysed as categorical (<42 mmol/mol, 42–47 mmol/mol, ≥48 mmol/mol) (<6.0%, 6.0–6.4%, >6.5%) BMI and waist‐to‐height ratio were analysed as continuous variables. Risk scores were analysed as ordinal categories (low, moderate, high, very high).

### 2.10 Statistical methods

Descriptive statistics summarised participant characteristics. Between group comparisons used *χ* ^2^ tests for categorical variables and *t*‐tests for continuous variables. Relative risk (RR) with 95% confidence intervals was calculated for DDP engagement. Logistic regression assessed predictive performance of BMI, waist‐to‐height ratio and risk scores, with ROC curves and AUC reported. Analyses were performed using SAS® and SPSS®.

### 2.11 Role of the funder

The funder of the study had no role in study design, data collection, data analysis, data interpretation, or writing of the report.

## 3 RESULTS

### 3.1 Baseline characteristics of participants

A total of 499 participants underwent HbA1c testing following recruitment through community pharmacies, and relevant data from 951 individuals with prediabetes identified during the study period by general practice were obtained from health records. Baseline characteristics are summarised in Table 1.

**TABLE 1:** Participant characteristics—community pharmacy and general practice.

Characteristic	Community pharmacy (%)	General practice (%)
Sex—Female	277 (55.5)	429 (45.1)
Age		
<21 years	2	0
21–29	17 (3.4)	5 (0.5)
30–39	39 (7.8)	18 (1.9)
40–49	54 (10.8)	64 (6.7)
50–59	92 (18.4)	189 (19.9)
60–69	170 (34.1)	312 (32.8)
70–79	103 (20.6)	251 (26.4)
80+	21 (4.2)	112 (11.8)
Missing	1	0
	499	951
SIMD		
1	174 (34.9)	148 (15.6)
2	138 (27.7)	168 (17.7)
3	76 (15.2)	201 (21.1)
4	69 (13.8)	245 (25.8)
5	37 (7.4)	156 (16.6)
Missing	5 (1.9)	31 (3.3)

Participants recruited through community pharmacies were younger than those identified through general practice (mean age 59.8 years [SD 14.2] vs. 65.5 years [SD 23.9]; *p* < 0.0001). The pharmacy cohort was also more socioeconomically deprived, with 63% residing in SIMD quintiles 1–2 compared with 33% of the GP cohort (*p* < 0.0001).

Women comprised 55.5% of the pharmacy group and 45.1% of the general practice cohort. Ethnicity reflected local population demographics, with 96.8% of participants in both cohorts identifying as White/White British. Among pharmacy participants, 16% reported current smoking and 40% were prescribed antihypertensive medication.

Mean BMI in the pharmacy cohort was 31.9 kg/m^2^ (SD 6.3), and mean waist‐to‐height ratio was 0.59 (SD 0.11).

**FIGURE 2:**
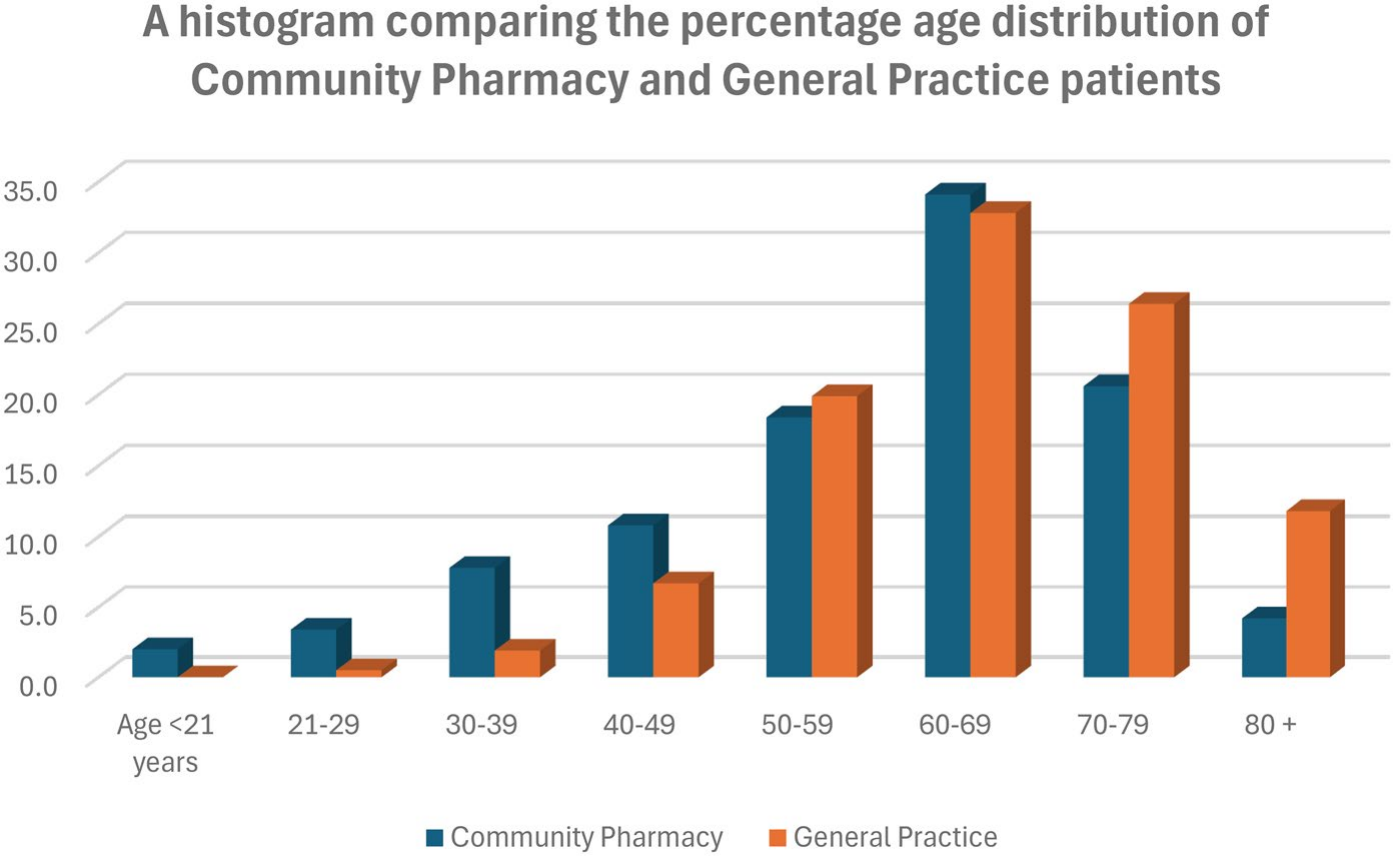
Pharmacy and general practice cohort comparisons—age distribution.

**FIGURE 3:**
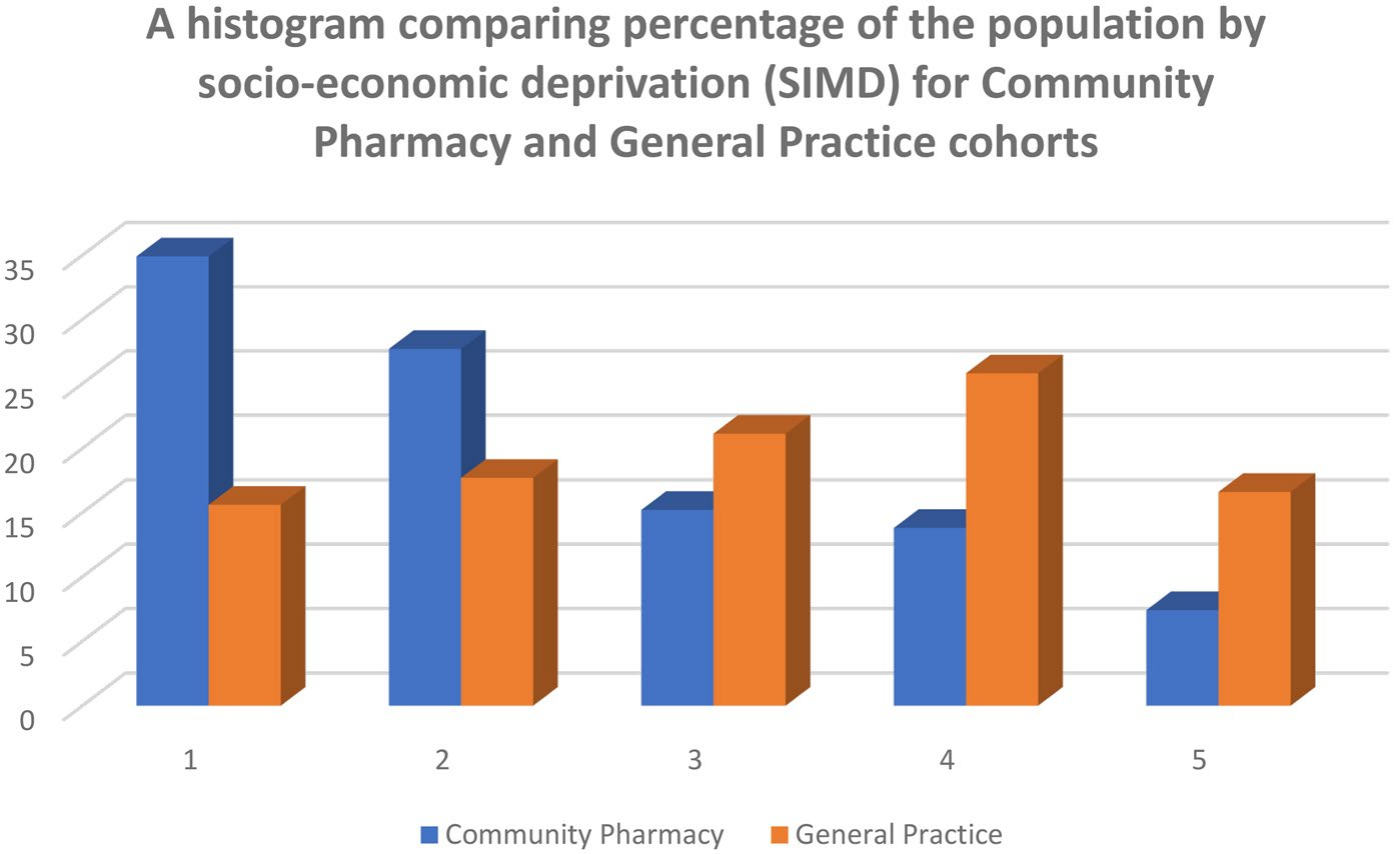
Pharmacy and general practice cohort comparisons—socio‐economic disadvantage. Scottish Index of Multiple Deprivation (SIMD).^23^

Sensitivity analyses restricted to participants with raised HbA1c (prediabetes or type 2 diabetes) showed that pharmacy‐identified participants remained significantly younger and more deprived than those identified through general practice (*p* < 0.0005 and *p* < 0.00001, respectively). Among pharmacy‐identified participants with prediabetes, 36% resided in SIMD quintile 1 and 63% in quintiles 1–2, compared with 16% and 33%, respectively, in the general practice cohort (Figures 2 and 3).

### 3.2 Assessment of participants for prediabetes and diabetes in community pharmacies

All 499 pharmacy participants received HbA1c testing. Of these, 75 (15%) had HbA1c values in the prediabetes range (42–47 mmol/mol) (6.0–6.4%) and 19 (4%) had HbA1c values ≥48 mmol/mol (6.5%), indicative of type 2 diabetes diagnosis.

Among pharmacy participants identified with prediabetes, 38 of 75 (51%) subsequently enrolled in the NHS Diabetes Prevention Programme (DPP). In comparison, among 951 individuals with prediabetes identified through general practice, 657 met age eligibility criteria (<70 years), of whom 212 (32%) enrolled in a DDP.

Pharmacy‐based identification was associated with a higher likelihood of DPP enrolment compared with the general practice route (relative risk 2.08, 95% CI 1.61–2.67). In absolute terms, this corresponded to 19 additional individuals per 100 eligible participants enrolling in a DPP through pharmacy referral.

Of the 19 pharmacy participants with HbA1c values of 48 mmol/mol (6.5%) or higher, 17 (89%) subsequently attended general practice and received a confirmed diagnosis of type 2 diabetes.

A further 405 pharmacy participants recorded HbA1c values <42 mmol/mol (6%) and were not referred for DDP.

### 3.3 Use of predictive risk assessment

Predictive risk assessment was undertaken in the pharmacy cohort using the *Know Your Risk* score. The mean score was 21 (SD 5.5), with 92.6% of participants classified as being at high or very high risk of diabetes (Table 2).

**TABLE 2:** Participant characteristics—community pharmacy assessment.

Characteristic	Number	Percent
Waist/height ratio		
<0.4	2	
0.4–0.49	48	9.6
0.5–0.59	181	38.3
0.6–0.69	144	28.9
0.7–0.79	43	8.6
0.8+	14	2.8
Missing	67	13.4
BMI		
<25	43	8.6
25–29	162	32.5
30–34	142	28.5
35–39	82	16.4
40–44	31	6.2
45–49	19	3.8
50–54	1	
55–59	1	
60–64	2	
Missing	16	3.2
Know your risk score		
0–9	10	2.0
10–15	26	5.2
16–19	176	35.3
20–24	156	31.3
25–29	88	17.6
30–34	34	6.8
35–39	4	
40–44	2	
Missing	7	1.4
HbA1c		
25–29 (4.4–4.8%)	5	1
30–34 (4.9–5.3%)	68	13.6
35–39 (5.4–5.7%)	252	50.5
40–44 (5.8–6.2%)	109	21.8
45–49 (6.3–6.6%)	24	4.8
50–54 (6.7–7.1%)	7	1.4
55–59 (7.2–7.6%)	1	
60–64 (7.7–8.0%)	2	
65+ (>8.1%)	6	1.2
Missing	25	5.0
Normal glycaemic range	404	81.0
Pre‐diabetic (42–47 mmol/mol) (6.0–6.4%)	75	15.0
Type two diabetic (>47 mmol/mol) (6.5%)	20	4.0

Receiver operating characteristic (ROC) analyses were used to evaluate predictors of raised HbA1c. In this cohort, BMI demonstrated the strongest discriminatory performance (AUC 0.72), compared with waist‐to‐height ratio (AUC 0.65) and Know Your Risk score (AUC 0.65), although no measure convincingly exceeded an AUC of 0.70 (Table 3).

**TABLE 3:** Predictive performance of measurements used to predict pre*d*iabetes.

Parameter	ROC curve (area)^SAS^	Significance^SPSS^
Body mass index	0.7231	<0.001
Waist circumference/height ratio	0.5331	0.731
Know your risk tool	0.6458	0.033

## 4 DISCUSSION

This study demonstrates that a community pharmacy–led pathway incorporating point‐of‐care HbA1c testing can identify individuals with previously unrecognised prediabetes and T2D and has the potential to support greater engagement with diabetes prevention interventions than standard general practice routes. Importantly, the pathway reached a population that was younger, more socioeconomically deprived and less well engaged with primary care, demonstrating that community pharmacies may offer a complementary access point for underserved populations to existing services rather than replicating current pathways of care.

A key and distinctive finding was the disproportionate benefit observed among individuals living in deprived areas. Participants identified through community pharmacies were substantially more likely to reside within the lowest SIMD quintiles than those entering care through general practice and were more likely to enrol in the Digital Diabetes Prevention Programme (DDP) once identified. Participants were also younger than those engaging with general practices, providing opportunities for earlier participation in preventative care. This suggests that the increased engagement observed may not simply be a function of enhanced referral processes, but rather reflects differential access, acceptability and trust associated with community pharmacies.^24^ Pharmacies may lower both practical and psychological barriers to engagement, including appointment availability, perceived stigma and competing priorities that disproportionately affect people living with socioeconomic disadvantage. However, while improved uptake is encouraging, this study did not examine downstream clinical outcomes or long‐term programme adherence. It therefore remains uncertain whether enhanced enrolment will translate into sustained risk reduction or reduced diabetes incidence in this population.

The identification of previously undiagnosed T2D in 4% of those tested was notable and higher than existing UK detection estimates (<2%).^25^ While this may reflect selection bias inherent in opportunistic testing, it also raises the possibility that current primary care‐based approaches fail to engage specific high‐risk groups until later in the disease trajectory. If the number of undiagnosed T2D in Scotland is around 45,000–55,000, evidence provided by this study supports the view of a disproportionate concentration in SIMD 1/2. This finding should be interpreted cautiously given the sample size, but it underscores the potential complementary value of community pharmacy testing as a case‐finding strategy, particularly in areas of high deprivation where delayed diagnosis is common.

The evaluation of triage strategies highlighted important limitations. Neither the *Know Your Risk* score nor waist‐to‐height ratio demonstrated sufficient predictive performance to meaningfully improve selection for HbA1c testing, and although BMI performed slightly better, no clear threshold could be defined. This is consistent with other comparisons which identify that the predictive performance of existing models is not satisfactory.^26^ These findings suggest that algorithmic or anthropometric triage alone will not be an efficient gatekeeper for prediabetes screening in real‐world pharmacy settings. Willingness to engage, perceived relevance of testing and opportunistic access may be equally important drivers of case detection and intervention uptake. Future work should consider. whether pragmatic approaches—such as targeting by age and deprivation or offering testing universally within defined settings—are more effective and equitable than increasingly complex risk stratification models.

### 4.1 Progression, sustainability and impact

For this model to have sustained impact within Scotland, progression beyond pilot delivery is required. Our study demonstrates the feasibility of implementing this model in a real‐world setting and our results suggest that such a model may enhance access to T2D prevention programmes for individuals with prediabetes, particularly for those living in areas with high socio‐economic deprivation; therefore, progression to a full‐scale evaluation of effectiveness is justified to inform service commissioning. For further progression, integration within existing NHS Scotland commissioning and contractual frameworks will be essential, alongside consistent funding for staff time, training, quality assurance and data integration.^26^ The extensive network of community pharmacies provides a clear mechanism for scale‐up, but implementation is likely to yield the greatest health gain with prioritisation of areas of high socio‐economic deprivation to maximise impact on health inequalities. Embedding the pathway within formal referral systems, with close integration to general practice, will be critical to ensure continuity of care and to avoid fragmentation.

The forthcoming transition to independent pharmacist prescribing in the UK offers a significant opportunity to strengthen this model further, including adding additional cardiovascular risk point‐of‐care measures. Pharmacist prescribers could support earlier clinical intervention, protocol‐driven management and more seamless escalation of care, potentially increasing both effectiveness and efficiency. However, progression will require robust evaluation of effectiveness and cost‐effectiveness, workforce capacity and opportunity costs compared with alternative prevention strategies.

### 4.2 Potential barriers to implementation

Several barriers may limit wider adoption. These include variability in pharmacy workforce capacity, competing service demands and the need for ongoing training and governance structures to support testing. Data sharing infrastructure between pharmacies and primary care remains inconsistent and may impede integration at scale. There is also a risk that unfunded service expansion could exacerbate workload pressures within community pharmacy, undermining sustainability. Finally, while pharmacies appear effective at reaching underserved populations, care must be taken to ensure that expansion does not inadvertently widen inequalities by concentrating services in areas with greater commercial or workforce capacity rather than greatest health need.

### 4.3 Comparison with international literature

Internationally, community pharmacy–based diabetes screening has been explored in several settings, including Australia, Japan and parts of North America.^27^, ^28^, ^29^ Similar to our findings, studies have shown that pharmacies can identify individuals at elevated type 2 diabetes risk who are not engaged with conventional healthcare services.^30^ However, a limitation of these interventions was that they relied on risk scoring alone or required participants to re‐engage with general practice for further assessment of glucose dysregulation prior to referral to DPP, leading to substantial attrition. In contrast, the present study demonstrates the feasibility of a direct test‐to‐care pathway, in which identification, prediabetes diagnosis and referral are integrated within the pharmacy setting. This approach may account for the higher intervention uptake observed and represents a substantive extension of prior work. Nevertheless, international evidence highlights similar challenges around sustainability, reimbursement and integration, reinforcing the need for system‐level support if pharmacy‐led prevention pathways are to achieve long‐term population health impact.^30^

### 4.4 Strengths and limitations

This study demonstrates that community pharmacies can identify people with prediabetes who are currently not known to primary care services and improve equality of access to those services. However, this study did not follow clinical outcomes of pathway engagement, only the relatively enhanced uptake of interventions by individuals currently missed by standard care. A further full‐scale experimental study is required to test the findings of this study and to generate evidence of effectiveness and health‐economic impact.

Missing data were minimal across variables. For anthropometric measures (BMI, waist‐to‐height ratio) and risk score components, missing values arose when participants declined measurement or when staff were unable to obtain accurate readings. Missing data were handled using complete‐case analysis, consistent with STROBE recommendations for feasibility studies. As missingness was low and showed no systematic pattern across age, sex, or deprivation quintiles, imputation was not undertaken.

## 5 CONCLUSIONS

In summary, the pathway successfully identified individuals being missed by standard routes and enhanced referral into available care pathways (prediabetes and T2D), possibly reflecting the influence of longitudinal positive relationships of socioeconomically deprived populations with pharmacy staff.

Our findings confirm the potential benefits of implementing disease prevention services into pharmacies to improve care of those in most need, simultaneously addressing current health inequalities of access to care, while showing the potential of this type of development to contribute capacity, along with enhanced clinical efficacy, to primary care services.

## AUTHOR CONTRIBUTIONS

Conceptualisation: Radley, Sutherland; Methodology: Radley, Beer, Farre, Rauchhaus, Thom, Sutherland; Data Curation: Beer; Formal Analysis: Radley, Beer, Rauchhaus; Investigation: Radley, Beer, Thom; Writing—Original Draft: Radley, Beer, Sutherland; Writing—Review & Editing: All authors; Supervision: Radley, Sutherland; Project Administration: Radley, Beer.

## FUNDING INFORMATION

This work was supported by the NHS Tayside Charitable Foundation. The funder had no role in study design, data collection, data analysis, data interpretation, or writing of the report.

## CONFLICT OF INTEREST STATEMENT

The authors declare no competing interests.

## ETHICS STATEMENT

This study was assessed by the Tayside Medical Science Centre Governance service and classified as a service improvement project, not requiring NHS REC review. Caldicott Guardian approval and service evaluation permissions were obtained. Staff interviews were approved by the University of Dundee REC (UoD‐SMED SLS‐Staff‐2024‐24‐43). All participants provided written informed consent.

## PATIENT AND PUBLIC INVOLVEMENT

Patients/public were not involved in study design, conduct, reporting, or dissemination.

## Supporting information


**Data S1.** STROBE Checklist for Cohort Studies.

## Data Availability

De‐identified data are available on reasonable request and subject to NHS Tayside governance approval.
