# Supplementary material for: Detection of prediabetes and undiagnosed type 2 diabetes in underserved communities using community pharmacy point‐of‐care testing: A prospective feasibility study
Source: Diabet Med. 2026 May 15;43(7):e70368. doi: 10.1111/dme.70368 (PMC13257908; doi:10.1111/dme.70368)
Supplement: Supplementary file 1 — Data S1. STROBE Checklist for Cohort Studies. [file DME-43-e70368-s001.docx]

# STROBE Checklist for Cohort Studies

Initial findings indicate that a Community Pharmacy Service directs previously undetected Pre-Diabetes and type 2 diabetes cases into care at a higher rate than current NHS pathways.

## Title and Abstract

| Item | Recommendation | Addressed |
| --- | --- | --- |
| 1a | Indicate the study design in the title or abstract | Addressed |
| 1b | Provide informative summary | Addressed |

## Introduction

| Item | Recommendation | Addressed |
| --- | --- | --- |
| 2 | Scientific background | Addressed |
| 3 | Objectives stated | Addressed |

## Methods

| Item | Recommendation | Addressed |
| --- | --- | --- |
| 4 | Study design presented | Addressed |
| 5 | Setting and dates described | Addressed |
| 6a | Eligibility and selection methods | Addressed |
| 6b | Matching | Not applicable |
| 7 | Variables defined | Addressed |
| 8 | Data sources and measurement | Addressed |
| 9 | Efforts to address bias | Addressed |
| 10 | Study size justification | Addressed |
| 11 | Quantitative variables handling | Addressed |
| 12a | Statistical methods | Addressed |
| 12b | Subgroups/interactions | Addressed |
| 12c | Missing data | Addressed |
| 12d | Loss to follow-up | Not applicable |
| 12e | Sensitivity analyses | Addressed |

## Results

| Item | Recommendation | Addressed |
| --- | --- | --- |
| 13a | Numbers at each stage | Addressed |
| 13b | Reasons for non-participation | Addressed |
| 13c | Flow diagram | Included |
| 14a | Participant characteristics | Addressed |
| 14b | Missing data | Addressed |
| 14c | Follow-up time | Not applicable |
| 15 | Outcome data | Addressed |
| 16a | Estimates with precision | Addressed |
| 16b | Category boundaries | Addressed |
| 16c | Absolute risk translation | Addressed |
| 17 | Other analyses | Addressed |

## Discussion

| Item | Recommendation | Addressed |
| --- | --- | --- |
| 18 | Key results summary | Addressed |
| 19 | Limitations discussed | Addressed |
| 20 | Interpretation cautious | Addressed |
| 21 | Generalisability | Addressed |

## Other Information

| Item | Recommendation | Addressed |
| --- | --- | --- |
| 22 | Funding and role of funders | Addressed |
